# Understanding the effects of a decentralized budget on physicians' compliance with guidelines for statin prescription – a multilevel methodological approach

**DOI:** 10.1186/1472-6963-7-68

**Published:** 2007-05-08

**Authors:** Henrik Ohlsson, Juan Merlo

**Affiliations:** 1 Social Epidemiology & HE, Department of Clinical Sciences in Malmö, Faculty of Medicine, Lund University, Sweden; 2 Unit of Health and Health Care Epidemiology, Department of Health and Health Care Management, Region Skåne, Sweden

## Abstract

**Background:**

Official guidelines that promote evidence-based and cost-effective prescribing are of main relevance for obvious reasons. However, to what extent these guidelines are followed and their conditioning factors at different levels of the health care system are still insufficiently known.

In January 2004, a decentralized drug budget was implemented in the county of Scania, Sweden. Focusing on lipid-lowering drugs (i.e., statins), we evaluated the effect of this intervention across a 25-month period. We expected that increased local economic responsibility would promote prescribing of recommended statins.

**Methods:**

We performed two separate multilevel regression analyses; on 110 827 individual prescriptions issued at 136 *publicly*-administered health care centres (HCCs) nested within 14 administrative areas (HCAs), and on 72 012 individual prescriptions issued by 115 *privately*-administered HCCs. Temporal trends in the prevalence of prescription of recommended statins were investigated by random slope analysis. Differences (i.e., variance) between HCCs and between HCAs were expressed by median odds ratio (MOR).

**Results:**

After the implementation of the decentralized drug budget, adherence to guidelines increased continuously. At the end of the observation period, however, practice variation remained high. Prescription of recommended statins presented a high degree of clustering within both publicly (i.e., MOR_HCC _= 2.18 and MOR_HCA _= 1.31 respectively) and privately administered facilities (MOR_HCC _= 3.47).

**Conclusion:**

A decentralized drug budget seems to promote adherence to guidelines for statin prescription. However, the high practice differences at the end of the observation period may reflect inefficient therapeutic traditions, and indicates that rational statin prescription could be further improved.

## Background

### Adherence to prescription guidelines

Prescription guidelines that promote evidence-based and cost effective prescribing of drugs are of main relevance for promoting effective and safe pharmacologic treatment as well as for the efficient use of a limited health care budget. Therefore, adherence to prescription guidelines has attracted considerable interest in many countries [1-3], including Sweden [4-6]. However, it is still insufficiently known to what extent guidelines from the drug committees are followed and the factors that at different levels of the health care condition prescription adherence to recommended medication [4,7,8].

In a previous study [9], we investigated the role of municipalities and outpatient Health Care Centres (HCCs) in understanding adherence to official guidelines on statin prescription in the county of Scania, Sweden. Using multilevel regression analysis we developed an epidemiological design suitable for monitoring practice variation and prevalence of adherence to guidelines along time. We noted that HCCs appeared to be more relevant than municipalities for understanding physicians' propensity to prescribe a recommended statin, and that the publication of the guidelines exerted a positive influence. In other words, prescription of recommended statins presented increasing trend and variance between HCCs and municipalities slightly decreased. However, at the end of the observation period the prevalence of adherence to guidelines was inappropriately low and practice variation unsuitably high, suggesting that inefficient therapeutic traditions were still influencing statin prescription. For this reason, it was suggested that more intensive interventions would be necessary to promote rational statin prescription.

### The decentralized pharmaceutical budget

The health services in Sweden are overwhelmingly tax-financed through county taxes [10]. Even if the Swedish Health Care System is rather homogenous all over the country, every of the 20 county councils in Sweden (Scania is one of the largest) have a high financial autonomy for managing health care services within their respective areas.

**Figure 1:**
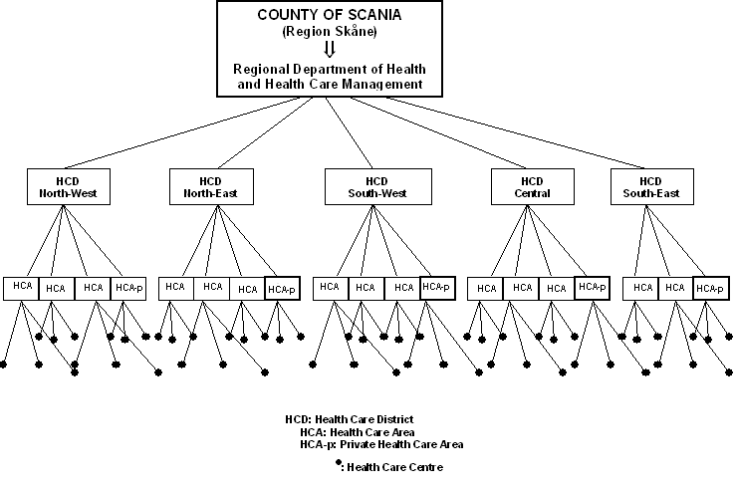
The structure of the health care system in the Scania County council.

In January 2004, the county council of Scania implemented a new system for managing the pharmaceutical budget. Under the new economic system, responsibility for the administration of the pharmaceutical budget passed from the regional Department of Health and Health Care Management to the 19 administrative Health Care Areas (HCAs) at five Health Care Districts (HCD) of the county [11]. See figure 1 for a short explanation of the structure of the health care system in the County of Scania. Simultaneously to the decentralized pharmacological budget, an information campaign was launched. In this campaign, specially trained pharmacists visited the HCCs and provided information on current local prescription patterns as a basis for reflection and prescription improvement. While the new economic system was compulsory, participation in the information campaign was voluntary.

### Aims of the study

In the present study we aimed to monitor and evaluate the effect of the decentralized pharmacological budget on prescribing behaviour and the role played by the different organizational levels (HCCs, HCAs and Health Care Districts) when it comes to understand physicians' adherence to prescription guidelines. Statins are an ideal medication group for this purpose, since they have very homogeneous indications and similar efficacy [12-14], which in principle eliminates the possibility of patient mix when comparing different practices and administrative areas.

A decentralized drug budget increases economic responsibility among prescribers by relocating control in management and decision-making from higher to lower levels of the health care organization and, thereby, it creates incentives for efficient drug prescription [15]. Therefore, our hypothesis was that the decentralized pharmacologic budget and would result in increased use of recommended statins and decreased variance between HCCs and HCAs throughout the 25-month observation period. While increased prevalence of adherence is the more informative parameter of a positive impact of the intervention, only a high prevalence in the area does not necessarily imply better care since it could depend of a few practices with a very high prevalence. Combining prevalence and variance measures we can obtain more complete information.

Due to the hierarchical structure of the data, we applied multilevel regression analysis (MLRA). MLRA accounts for and informs about the dependence of the outcome within organizational levels, and thereby not only produces accurate statistical estimations but also generates information regarding patterns of variation at different levels – an aspect of high relevance for investigating therapeutic traditions [16,17].

## Methods

### The register of pharmacological agents

We obtained information from the Swedish National Prescription Register [18], administrated by the Swedish Corporation of Pharmacies and based on record of sales. While the decentralized budget started in January 2004, the current recommendations for drug prescription were introduced in March 2004 and so did our observation period. During the 25-month period between March 2004 and March 2006 we selected all 110827 prescriptions of statins issued by public physicians and all 72012 prescriptions issued by private physicians at 136 public and 115 private HCCs in 14 public and 5 private HCAs in Scania. Statins were defined according to the Anatomical Therapeutical Chemical (ATC) classification system code C10AA [19].

A small percentage (6%; 12 683/195 522) of prescriptions were excluded since they had unidentified origin, they were from places outside Scania, or from HCCs with sporadic statin prescription (i.e., less than 50 prescriptions) during the observation period.

Each prescription, regardless of number of drugs, has a unique serial number in the register. The register data includes information about the age and gender of the patient, the health care facility where the prescription was issued, the brand name and ATC code for both prescribed and dispensed drugs, and whether the prescription was an initial or a repeat prescription.

For descriptive purposes we expressed statin utilization as direct age-standardized number of defined daily doses (DDD)/1000 inhabitants/day obtained by the Equivalent Average Rate methodology [20,21].

This study is a part of the LOMAS project (Longitudinal Multilevel Analysis in Scania) [22] that was reviewed and approved by the Swedish Regional Ethical Review Board in Lund.

### Individual-level variables

At the individual level, the outcome variable was *prescription of simvastatin *(yes vs. no). Simvastatin (regardless of brand, but excluding the original brand ZOCORD^®^) was the recommended statin in Scania during the whole observation period. Simvastatin has proved efficacy [12-14,23] and is the cheapest statin in Sweden. Though a prescription is valid for one year, the reimbursement system accepts a maximum of three months' supply per dispensation, so we selected initial prescriptions only in order to reduce the risk of counting the same prescription more than once.

Individual *age *in years was centred on the mean of 67 for prescriptions issued at public practices, and 66 for those issued at private practices. *Sex *(men vs. women) was defined by a dummy variable. *Time *(in months) was a continuous variable; March 2004 was recoded as 0, April 2004 as 1, and so on to March 2006 that was recorded as 24. Therefore, March 2004 was the intercept value in the regression analysis.

### Area-level variables

#### The structure of the health Care System at the county of Scania

The Region of Scania is situated on the southern part of the Scandinavian Peninsula. The county is geographical divided into 33 municipalities and its area covers less than 3% of Sweden's total area. The population of about 1.2 million represents, however, 13% of Sweden's total population. At the time of this study the health care system at the county of Scania was organized into five health care districts (northwest, northeast, southwest, southeast, and central). These five health care districts managed 19 administrative HCAs which, in turn, controlled 251 HCCs (Figure 1). Of those HCCs, 136 were public administered primary health care centres and hospital outpatient care clinics, and 115 were private primary HCCs assisted by private general practitioners (GPs) and other private specialists. Only five of the 19 HCAs managed private HCCs. The remaining 14 HCAs managed only public HCCs; nine HCAs managed only outpatient clinics at large public hospitals while five administered primary health care centres.

#### Participation in the information campaign

Simultaneously to the introduction of the decentralized budget, an *information campaign *for supporting appropriate prescription at the HCCs was carried out through the entire observation period. Participation in this campaign was voluntary. Since the campaign could influence prescription patterns independently of any possible effect of the decentralized budget, we included a variable indicating whether the HCC participated in the information campaign or not.

#### Budget decentralization at the HCA level and HCCs with own budget administration

In the new compulsory system of decentralized pharmaceutical budget, the responsibility for the administration of the pharmaceutical budget was transferred from the regional Department of Health and Health Care Management to every of the 19 administrative HCAs. However, nine of the 14 publicly-administered HCAs decided to implement a more intense decentralization by transferring the budget responsibility to their HCCs. Since this circumstance could influence prescription patterns, such HCCs were identified by a dummy variable. In the analyses, HCCs without their own budget management were used as reference in the comparisons.

#### Percentage of prescriptions from specialist physician at the HCC level

Since proximity to specialized care and the particular knowledge that it conveys might influence adherence with prescription guidelines, we also identified those HCCs which employed *specialist *physicians other than GPs. In the analyses, HCCs employing GPs alone were used as reference in the comparisons.

### Multilevel logistic regression models

We used *multilevel logistic regression analysis *to estimate the probability of prescribing a recommended statin, while accounting for the hierarchical structure of the data (i.e., patients nested within HCCs nested within HCAs) represented in figure 1[16,17].

Since physicians working in private practices may be less receptive to the policies of the county council than those working in public facilities, and because this might modify the effect of the decentralized budget, we performed our analyses separately for physicians under public or private administration

Public HCCs and HCAs were included in the analysis as random terms. However, because there were only five health care districts, which is a low number for including health care districts as a random term, they were included as a dummy variable (i.e., fixed effects), using the southwest district as reference in the comparisons. Since there was only one private HCA in each health care district, in the multilevel analysis of privately administered health care the only random term was HCC.

We developed three consecutive models. Model A included the area (i.e., HCA and HCC) random parameters together with *time*. The intention of this model was to investigate temporal trends of prescription of recommended statins throughout our observation period. Model B included the individual covariates age and sex. Finally, model C added the area-level variables; health care districts, information campaign, presence of specialist physicians other than GPs, and HCC with own budget responsibility. In this way we could investigate whether these contextual characteristics explained residual variation at the HCC and HCA levels.

In the fixed-effects part of the multilevel analysis, we calculated odds ratios (OR) and their 95% confidence intervals (95% CI) from the regression coefficients and their standard errors.

In the random-effects part of the multilevel analysis, we obtained the *variance *(*SE*) at the HCC and HCA levels. We calculated the *proportional change in variance *(PCV) between two consecutive models [24]. We also allowed the regression coefficients of the variables *time *and *sex *to be random at the HCC level (i.e. random slope analysis) in order to investigate whether these individual-level associations varied between different HCCs. In the presence of slope variance, the HCC variance becomes a function of the *individual variables*. We calculated the variance function as described elsewhere [25].

Theoretically the concept of intraclass correlation (i.e., the percentage of the total variance that is at the area level) is an intuitive measure of therapeutic traditions [9,26,27]. However, in multilevel logistic regression models, the fact that the variances at the area and at the individual levels are measured on different scales makes it difficult to interpret the intraclass correlation. Therefore, we calculated the median odds ratio (MOR) [28,29]. The MOR translates the variance into the widely used OR scale, and can thereby be directly compared with the ORs of individual or area variables. In very simple terms, the MOR could be interpreted as how much a physician's probability of prescribing a recommended statin would (in median) increase if this physician moved to a HCC/HCA with higher adherence to guidelines. A MOR of 1 indicates that there are not differences between HCCs/HCAs in the probability of prescribing a recommended statin. The larger the differences between HCCs (or HCAs) are, the larger the MOR will be.

Even if the overall OR for the association between an area (i.e., HCC or HCA) variable and the outcome is conclusively higher or lower than one, the distribution of OR for pairwise comparison between exposed and unexposed areas could contain a considerable percentage of ORs of opposed direction. Therefore, we calculated the percentage of ORs of opposed direction as complementary information to the overall OR of each area-level variable. This index considers the area residual variance in the calculation of the ORs of the area level variables, and indicates the extent to which the area variable under study is of importance as compared with residual area variations. If the index is 50% the association has no relevance. Details of the formulas and an extended explanation of the statistical analysis can be found elsewhere [30]. An Excel spreadsheet with formulas is available on request.

### Ranking of outpatient health care centres and administrative health care areas

Following previous recommendations for comparing performance between different health care units [31], we ranked HCCs and HCAs according their posterior means (also known as "shrunken residuals") obtained from the multilevel regression analyses. Each residual corresponds with the OR of adherence with guidelines (logarithmic scale) of the unit, with the whole county as reference in the comparisons.

Parameters were estimated by MCMC methods [32] and the goodness of fit was evaluated using the deviance information criteria (DIC). We used the MLwiN 2.02 software developed by Goldstein's research group [25].

## Results

The age-standardized utilization of statins in the whole county increased from 131 DDD/1000 inhabitants/day in 2004 to 177 in 2006, and a similar increasing trend was observed in all health care districts. Throughout the whole observation period, prescription of statins was highest in the northwest district and lowest in the central district (Table 1).

**Table 1:** Characteristics of the initial prescriptions of statins issued in Scania, Sweden, between March 2004 and March 2006, by Health Care District and specifying prescription of simvastatin (i.e., recommended drug).

	Whole Scania		North-West		North-East		Central		South-West		South-East	
	All	Simvastatin	All	Simvastatin	All	Simvastatin	All	Simvastatin	All	Simvastatin	All	Simvastatin

Number of prescriptions (Public HCCs)	110,827	68,372 (62%)	27,562	17,138 (62%)	19,145	12,978 (68%)	27,509	15,932 (58%)	28,373	16,264 (57%)	8,328	6,060 (74%)
Number of prescriptions (Private HCCs)	72,082	36,256 (50%)	20,270	9,958 (53%)	7,801	4,656 (64%)	7,922	4,342 (58%)	34,024	13,224 (42%)	7,272	4,076 (60%)
Men Public/private	56/55	55/55	53/55	53/55	55/55	55/56	58/52	57/52	56/55	56/55	57/55	57/54
Mean age in years (Public/private)	67/66	67/65	67/67	67/66	68/68	67/68	67/65	67/65	66/66	66/66	68/67	68/67
Number of HCAs (Public/private)	14/5		3/1		3/1		3/1		3/1		2/1	
Number of HCCs (Public/private)	136/115		29/28		25/13		36/18		39/41		7/15	
Percentage of prescriptions from HCCs that participated in information campaign (Public/private)	82/22	82/28	98/24	97/27	97/80	97/80	100/47	100/50	33/0	31/0	100/34	100/36
Percentage of prescriptions from HCCs with own budget administration (Public)	71	71	83	81	87	86	91	89	38	42	35	36
Percentage of prescriptions from specialist physician (Public/private)	34/37	37/34	29/38	32/33	32/4	35/2	35/41	37/43	38/48	42/46	35/15	36/20

**DDD/inhabitants/day for statins**												

DDD/inhabitants/day, 2004	131		153		127		110		132		129	
DDD/inhabitants/day, 2005	152		180		147		126		152		154	
DDD/inhabitants/day, 2006	177		207		171		147		178		186	

HCC = outpatient health care centre.

Table 1 shows that the mean age of the patients receiving a statin prescription was 67 years in the public sector and 66 years in the private. Overall, men were prescribed statins more often than women. More statin prescriptions were issued from public HCCs than from private HCCs. Of all the statin prescriptions issued at public HCCs, 82% originated from a HCC participating in the information campaign, but this figure was only 22% for private HCCs, and varied greatly between districts, being lowest in the southwest district. More statins were prescribed by HCCs composed of GPs alone than by HCCs including other specialists. Of the statin prescriptions issued at public HCCs, 71% originated from HCCs that managed their own pharmacologic budget.

**Figure 2:**
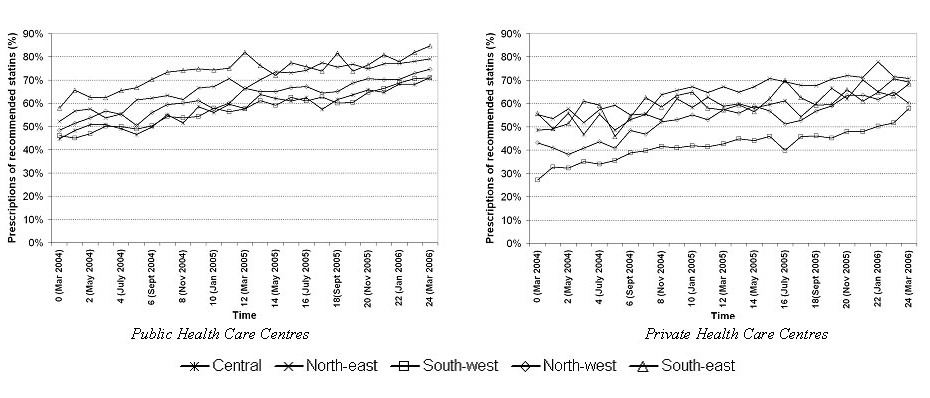
Percentage of recommended statins among initial statin prescription in the health care districts of the county of Scania, public health care centres (right) and private health care centres (left).

Overall, the prevalence of guideline adherence was 62% in the public sector and 50% in the private, with a clear increasing trend during the whole study period. In the first month, 48% of the public and 39% of the private HCCs prescribed recommended statins, and this percentage increased to 74% in the public and 62% in the private sector by the end of the study period. These trends were similar in all five health care districts, but adherence was always lowest in the southwest district, and highest in the northeast and southeast districts (Figure 2).

In model A (see Table 2), the MOR_HCA-HCC _in the public sector was 2.28 indicating that a physician's median probability of prescribing a recommended statin would approximately double if this physician moved to an HCC in an HCA with greater adherence to guidelines. However, when decomposing the MOR in specific levels, the propensity of prescribing recommended statins presented a higher degree of clustering at the HCC level than at the HCA level (MOR_HCC _= 2.18 vs. MOR_HCA _= 1.31). The MOR_HCC _in the private sector was 3.47, indicating an even stronger clustering among private HCCs.

**Table 2:** Multi-level logistic regression analysis of adherence to statin prescription guidelines in the county of Scania, Sweden

	**Model A**		**Model B**		**Model C**		
	Public	Private	Public	Private	Public	Private	

**Fixed effects**	OR (95% CI)	OR (95% CI)	OR (95% CI)	OR (95% CI)	OR (95% CI)	OR (95% CI)	
Time	1.05 (1.04–1.05)	1.06 (1.05–1.07)	1.05 (1.04–1.05)	1.06 (1.05–1.07)	1.05 (1.04–1.05)	1.05 (1.04–1.07)	
Time^2		1.00 (1.00–1.00)		1.00 (1.00–1.00)		1.00 (1.00–1.00)	
Sex (women vs men)			0.93 (0.87–0.98)	0.92 (0.84–1.01)	0.93 (0.88–0.99)	0.92 (0.85–0.99)	
Age (one year increase)			1.000 (1.000–1.000)	1.00 (1.00–1.00)	1.00 (1.00–1.00)	1.00 (1.00–1.00)	
Information campaign (Yes vs No)					1.11 (0.90–1.39)	1.46 (0.73–2.34)	
% opposed ORs					46%	42%	
Specialist physician vs GP					1.41 (1.18–2.01)	0.97 (0.66–1.31)	
% opposed ORs					38%	49%	
HCC with own budget administration (yes vs No)					0.82 (0.68–1.06)		
% opposed ORs					43%		
North-West health care district			–	–	1.37 (1.01–1.93)	1.66 (1.09–2.50)	
% opposed ORs					39%	39%	
North-East health care district			–	–	1.39 (0.84–2.04)	1.78 (1.04–3.89)	
% opposed ORs			–	–	39%	38%	
South-West health care district			–	–	Reference	Reference	
South-East health care district			–	–	2.03 (1.08–3.92)	1.46 (0.84–1.99)	
% opposed ORs			–	–	27%	42%	
Central health care district			–	–	0.85 (0.47–1.15)	1.53 (1.10–2.73)	
% opposed ORs			–	–	45%	41%	

**Random effects**	Variance (95% CI)	Variance (95% CI)	Variance (95% CI)	Variance (95% CI)	Variance (95% CI)	Variance (95% CI)	**PCV**

HCA (intercept)	0.08 (0.01 – 0.38)		0.15 (0.04 – 0.42)		0.04 (0.00–0.17)		50%
MOR_HCA_	1.31 (1.11 – 1.80)		1.44 (1.22 – 1.87)		1.21 (1.04 – 1.49)		
HCC (intercept)	0.67 (0.51 – 0.89)	1.70 (1.28 – 2.32)	0.62 (0.46 – 0.84)	1.80 (1.34 – 2.44)	0.62 (0.47 – 0.82)	1.71 (1.26 – 2.34)	8% (Pu)
MOR_HCC_	2,18 (1.98 – 2.46)	3.47 (2.94 – 4.28)	2,12 (1.92 – 2.39)	3.60 (3.01 – 4.43)	2.12 (1.92 – 2.37)	3.48 (2.92 – 4.31)	0% (Pr)
OHC and HCA (intercept)	0.75		0.77		0.66		12%
MOR_HCA-HCC_	2.28		2.31		2.17		
Time (slope)	0.000 (0.000 – 0.001)	0.001 (0.001 – 0.002)	0.000 (0.000 – 0.001)	0.001 (0.001 – 0.002)	0.000 (0.000 – 0.001)	0.001 (0.001 – 0.002)	
Sex (slope)	–		0.063 (0.043 – 0.093)	0.149 (0.103 – 0.214)	0.063 (0.043 – 0.094)	0.147 (0.102 – 0.214)	
Deviance information criteria (DIC)	136 649.6	87 811.1	136 114.6	87 421.9	136 113.9	87 422.4	

HCC = outpatient health care centre. MOR = median odds ratio. OR = odds ratio.

95% CI = 95% credible interval. SE = standard error.

PCV = proportional change in variance (PCV) in model C using model A as reference

**Figure 3:**
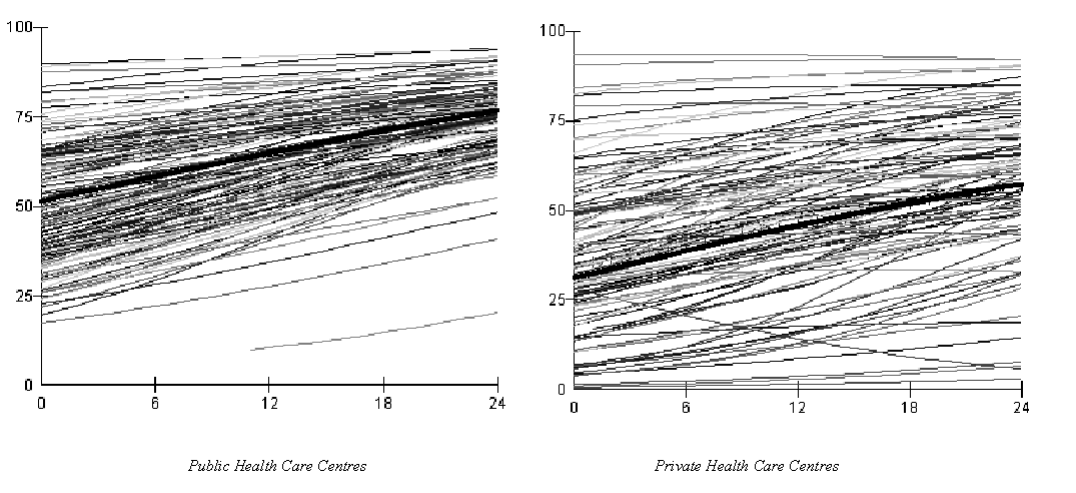
Predicted probabilities for prescribing recommended statins at public (left) and private (right) health care centres in Scania.

As illustrated in Figure 2, there was an increasing temporal trend in prescription of recommended statins. However, this trend differed between HCCs, and the *time *variable presented a significant slope variation between HCCs evidenced by the plot of the predicted values in Figure 3. Because of this slope variance, the MOR_HCC _became a function of time indicating that even if the MOR_HCC _decreased throughout the study period, the final variation was still high (MOR_HCC _= 1.86 in the public sector and 2.73 in the private).

We did not observe any significant slope variation of the time variable at the HCA level.

The analysis of the PCV in Table 2 indicates that 50% of the differences between HCAs were explained by the individual and contextual characteristics included in model C. In relation to variance between HCCs, this percentage was only 8% in the public sector and 0% in the private. The DIC diagnosis suggested that model C represent an improvement over model B in goodness of fit for the public sector, but not for the private.

**Figure 4:**
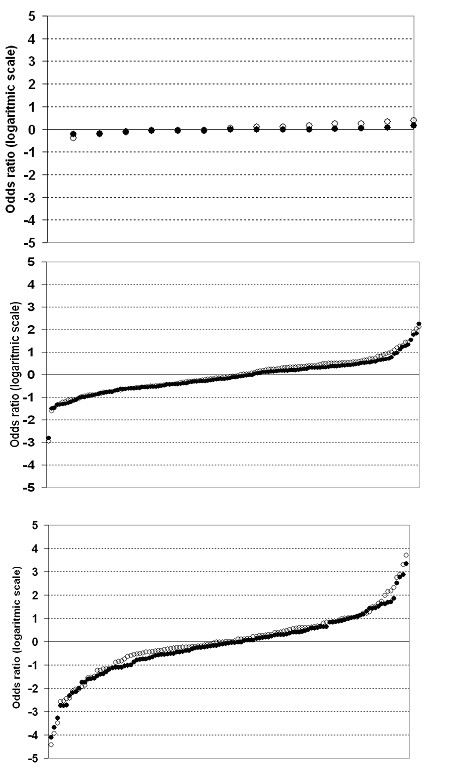
Differences (i.e. residuals) between health care centres obtained from the model including random parameters together with time (unfilled circles) and the model also including age, sex, health care districts, information campaign, presence of specialist physician other than general practitioners, and degree of decentralization (filled circles). Public administrative health care areas (top), public health care centres (middle), and private health care centres (bottom).

The ranking of the HCCs and HCAs regarding the prevalence of prescription of recommended statins in each area relative to the overall prevalence in the county at the beginning of the study period is presented in Figure 4 both before (model A) and after (model C) making adjustments. The differences between HCAs disappeared after adjustment, but although many HCCs changed position in the ranking, the HCC dispersion around the mean was not reduced after adjustments.

Overall, men had higher use of statins (Table 1) but lower probability than women of being prescribed a recommended statin (Table 2). The existence of slope variation, however, indicated that this pattern of association was not constant in all HCCs.

Table 2 shows that the probability of prescribing recommended statins was not conditioned by participating in the information campaign. Also, our results suggest that even if adherence to guidelines clearly improved after the implementation of the decentralized budget, this improvement was not more intense among HCCs with own budget administration. Actually – even if the results were not conclusive at the 95% level – comparing to HCC without own budget administration those with own economical responsibility presented a lower, rather than higher, probability of adherence with guidelines, OR = 0.82 (95%CI: 0.68–1.06),

Among the public HCCs, prescriptions of recommended statins were more frequently issued at HCCs with specialist physicians other than GPs, but no such association was observed for private HCCs. Compared with the southwest district, all other districts presented a higher probability of prescribing recommended statins for both public and private HCCs, except public HCCs in the central district.

DiscussionIn this study we evaluated the effect of a decentralized pharmaceutical budget intended to promote adherence with prescription guidelines. According to our results, this intervention appeared to considerably improve adherence to guidelines for statin prescription and, promoted efficient pharmacological treatment.

We performed separate analyses for publicly and privately administrated HCCs, since we believed that the administrative background could modify the effect of these interventions. However, even though guideline compliance was systematically lower among private facilities, compliance in both the public and the private sector increased progressively from the implementation of the decentralized budget through the observation period.

Since our study is observational, several bias and confounding factors need to be considered. While face-to-face visits such as those performed during the intervention campaign have a documented effect on prescription patterns [33], in our analysis participation in the information campaign was not associated to higher adherence with guidelines. Given that participation in this campaign was free, it is probable that other reasons apart from the information campaign itself confound the observed association. For example, HCCs with a very low adherence to guidelines at the start of the intervention may be especially prone to participate in order to improve their prescription patterns. This effort would have only raised adherence to the same level as rest of the HCCs. Due to selection biases interpretation of the effect of the information campaign is limited.

Evaluation of the decentralized budget was less affected by bias since the adoption of the new budget system was obligatory and embraced all the prescribers in the county. Nevertheless, it cannot be ruled out that other external influence besides the decentralized budget could provide an alternative explanation of our results. Also, as we shown in our previous article [9], adherence with guidelines was slightly increasing before the implementation of the decentralized budget. Nevertheless, it is reasonable to believe that the intense trend of increasing prescription of simvastatin occurring after the implementation of the budget actually reflects the new economic responsibility of the prescribers. Several studies suggest that payment method affects physicians' prescription behaviour [2,33,34]. Moreover, even if guideline dissemination alone has a less important effect on prescribing patterns [9,33,34], it has proved to be effective as part of a multifaceted intervention and as a predisposing foundation for other strategies.

Observational studies are often the only option for investigating questions that for reasons of feasibility, costs, or ethics cannot be analysed by randomized trials [35,36]. In our observational study we used multilevel regression analysis, which not only produces more correct statistical analysis (i.e., it accounts for residual correlation within areas) but also informs about the role that different health care levels play in understanding drug prescription and utilization [16,17].

Our results suggest that adherence to guidelines seemed to be conditioned by contextual factors, especially at the HCC levels. Based on the MOR measure, we observed that physicians from the same HCC and from the same HCA exhibited a similar propensity to prescribe simvastatin. This clustering of prescription behaviour was greater at the HCC than at the HCA level, which suggest that interventions directed at the HCC level would in principle be more effective than those directed at the HCA level. Also, private HCCs had both greater clustering of prescription behaviour and lower adherence to guidelines, suggesting that interventions directed at private HCCs could be appropriate.

In a previous multilevel analysis [9] we investigated HCCs nested within municipalities rather than within HCAs as in the present study. However, since HCAs are responsible for the management of the new decentralized budget, we did not consider the municipality as a relevant level in this investigation, and a sensitivity analysis (data not shown) including the municipality level confirmed our assumption. In the present study we did not have access to information at the physician level, but two previous studies have shown that variations at the physician level accounted for about 50% of variations at the HCC level [37,38].

The variation between public HCAs was very low and could partly be explained by contextual characteristics such as health care district, participation in the information campaign, the presence of specialist physicians other than GPs, and degree of decentralization. Contrarily, the variation between both public and private HCCs was very high and remained unexplained throughout the whole observation period (i.e., model C in Table 2). Practice variation between HCCs is a common phenomenon that does not necessarily need be inappropriate, but rather may reveal different strategies for confronting a specific therapeutic problem. Practice variation might reflect medical uncertainty resulting from differences in information and knowledge. However, when the same pharmacological therapy is available as different brands at different prices and the prescriber selects the more costly, there are reasons to question the suitability of the observed practice variation [26,27,39-43]. In this context and since all statins have the same indication and only marginal differences in efficacy, there are no solid reasons for justifying the prescription of expensive brands in general and for some patients rather than others in particular [12-14].

The process of prescription includes a number of phases (identification of the health problem, decision to prescribe, choice of medication, decision to cease using a specific therapy) and could be influenced at different levels (e.g. at the level of the patient, prescriber, HCC, HCA, or health care district). However, few studies have aimed to understand the relative importance of these different levels [7,26,27,37,44]. Moreover, even if adherence with guidelines in general is a well-developed research topic [1-3], as far we know only to investigations have been focused on adherence to guidelines of statin prescription, [45,46] and only our current and previous work has applied multilevel regression analyses [9]. The present investigation provides valuable and original information that could be of relevance for planning and evaluating interventions aimed to promote efficient and evidence-based prescription.

Because of similar indications and efficacy, statins are an ideal medication group for investigating prescribing behaviour. For this reason, there is no rationale for considering patient characteristics as confounding factors when investigating practice variation. Rather, the value of including individual variables resides in the understanding of the factors that condition adherence to prescribing guidelines. In the present investigation we only considered basic individual variables such as age and gender. An extended study of determinants of adherence to guidelines may also require the investigation of the influence of socioeconomic differences of the patients on the process of prescribing as well as applying qualitative research methodology [47].

According to the prescription guidelines in the county of Scania there is one patient related condition in which a non-recommended statin has a preferential indication [14]. In fact, when simvastatin does not reach sufficient effect, a change to atorvastatin 80 mg is officially recommended. However, a complementary analysis indicates that atorvastatin 80 mg was only 0.5% of all the statin prescriptions, and including this substance in the category of recommended statin had no influence on the results.

In a previous study of ours performed in the county of Scania, the basal level of adherence with recommended statins was much lower than in the present investigation. The main reason for this difference is that in the previous study period, guidelines were very strict, including only Pravachol and Simvastatin GEA rather than Simvastatin as in the present investigation. Certainly, the existence of plain guidelines facilitates adherence, but it does not influence the conclusions of the present investigation.

Our empirical analysis found that men were prescribed more statins than women, but women had a slightly higher probability than men of being prescribed the cheaper, recommended, statins. Men have a higher prevalence of ischemic heart disease and they are therefore expected to be more represented among statin users. On the other hand, the gender differences in the prescription of simvastatin did not seem rational. It is possible that qualitative analyses would give more information on the reasons for this prescribing behaviour.

It is known that some non-recommended statins like rosuvastatin have been the subject of safety concerns [48-50], which may have promoted prescription of simvastatin beyond the influence of the guidelines. However, if this is true, this external influence simultaneously affected all the HCCs and HCAs and therefore should have had less relation to variance between HCCs and HCAs.

Multilevel regression analyses are a very suitable methodology for studying practice variation, and are being successfully employed in an increasing number of studies in the field [7,9,26,27,37,44,51]. They are a useful epidemiological tool for investigating and quantifying medical practice variation, and for evaluating and planning interventions.

## Conclusion

In conclusion, the decentralized pharmaceutical budget seems to considerably influence prescription behaviour and increase adherence to guidelines for statin prescription. Though, at the end of the observation period, variation between HCCs was still high, especially among private HCCs. These remaining disparities may reflect inefficient therapeutic traditions, and suggest that more intensive interventions may be necessary to promote adherence to prescription guidelines [52]. Obviously, a decentralized pharmaceutical budget [11,15] transfers power in management and decision-making from higher to lower levels of the health care organization, which in turn increases economic responsibility among prescribers and creates incentives for efficient drug prescription. Therefore, as a natural consequence, adherence to the drug committee's recommendations increases.

## Competing interests

The author(s) declare that they have no competing interests.

## Authors' contributions

HO and JM developed the original idea, participated in the design, analysis and drafted the manuscript. HO carried out the statistical analysis. All authors read and approved the final manuscript.

## Pre-publication history

The pre-publication history for this paper can be accessed here:


